# Confirmatory factor analysis of the Children's Eating Behaviour Questionnaire in a Chinese urban preschooler sample

**DOI:** 10.1186/s12966-021-01175-y

**Published:** 2021-09-03

**Authors:** Nan Zhou, Luning Sun

**Affiliations:** 1 College of Early Childhood Education, Capital Normal University, #5, North 3rd Street, Fu Cheng Rd., Haidian District, Beijing, 100048 China; 2 The Psychometrics Centre, Cambridge Judge Business School, University of Cambridge, Trumpington Street, Cambridge, CB2 1AG UK

**Keywords:** Eating behaviours, Chinese preschoolers, Validity

## Abstract

**Background:**

Understanding young children's eating behaviours is vital to childhood obesity prevention. However, the widely used Children's Eating Behaviour Questionnaire (CEBQ) has not been validated in Chinese young children. Thus, the present study aimed to assess the validity of the CEBQ in a Chinese urban sample of preschool children.

**Methods:**

Participants included 389 mothers with preschool children residing in Beijing, China. Confirmatory factor analysis was conducted, and measurement invariance between child genders was evaluated.

**Results:**

The modified 8-factor structure of the CEBQ exhibited acceptable model fit in our sample, and no measurement bias against any gender was observed. The associations between the CEBQ factors and child age showed that desire to drink, emotional overeating, and emotional undereating significantly decreased with age, but food responsiveness increased with age. The relation between child BMI and the CEBQ factors provided convergent validity for the CEBQ.

**Conclusions:**

Our study supported the validity of the CEBQ as a measurement tool for examining preschool children's eating behaviours in a Chinese urban sample.

## Background

Childhood obesity is a serious public health concern globally. During the past decades, China has experienced dramatic socio-economic changes and nutritional transitions [1]. The prevalence of childhood overweight and obesity increased from 8.2% in 1991 to 18.3% in 2006, especially among the urban population [2]. In 2005, the combined rate of childhood overweight and obesity is 36.8% in north coastal high-SES cities (e.g., Beijing, Shanghai).

Urban children in China are more likely to be exposed to an obesogenic environment and consume larger amounts of unhealthy snacks and fried foods, compared with their rural counterparts [2, 3]. There is strong empirical evidence that poor eating behaviours and dietary preferences are established early in childhood [4, 5], and individual differences in appetite and eating behaviours emerge since infancy [6]. For instance, children's satiety responsiveness, slowness in eating, and food fussiness are negatively associated with their weight, whereas food responsiveness, enjoyment of food, emotional overeating, and desire to drink are positively associated with child weight [7]. Thus, understanding young children's eating behaviours is vital to childhood obesity prevention in the understudied Chinese urban population.

### Children's Eating Behaviour Questionnaire

The Children's Eating Behaviour Questionnaire (CEBQ) is a widely used parent-rated questionnaire that assesses eight subscales of child eating behaviours, including satiety responsiveness, slowness in eating, food fussiness, food responsiveness, enjoyment of food, desire to drink, emotional undereating, and emotional overeating [8]. Extensive evidence has shown that the CEBQ established good internal consistency, test-retest reliability, and construct validity empirically [8–10].

The CEBQ was originated in the UK, and has been translated to many languages. When being used in different languages and/or populations, inconsistent factor structures have been reported. For instance, during the development, Wardle and colleagues discovered two solutions, one with 8 subscales and the other with 7, combing satiety responsiveness and slowness in eating into one factor [8]. The original 8-factor structure showed acceptable model fit for 6-to- year-old Chilean children [11]. Also, a re-specified 8-factor model provided an acceptable model fit for 1-to-5 year-old Australian children, and reasonable fit for both Indian and Chinese immigrant children in Australia [12]. However, the factor structures failed to replicate in other studies, and modified factor structures have been suggested in different cultures. For example, the original 7-factor structure in Wardle et al. showed poor model fit in low-income ethnically diverse 2-to-5-year-old American children [8, 13]. A revised 7-factor structure, with satiety responsiveness clustered with emotional overeating, exhibited acceptable fit for 6-to-7-year-old Dutch children and 1–to-6 year-old Swedish children [14, 15]. A 6-factor structure provided acceptable model fit for 3-to-13-year-old Portuguese children [16].

While most of the studies mentioned above focused on Western samples, only a few studies validated the CEBQ in Asian samples. Quah and colleagues conducted exploratory factor analysis and revealed a 7-factor structure, with food responsiveness merged with enjoyment of food and emotional overeating, for 3-year-old children in multi-ethnic Singaporean sample [17]. Cao and colleagues found a 7-factor solution for Chinese 12-to-18 month-old infants, in which food responsiveness was split into two factors and satiety responsiveness and enjoyment of food were not detected [18]. Thus, validation of the CEBQ in Chinese urban preschool children that is currently scarce in the literature is greatly needed.

Both principal component analysis and exploratory factor analysis have been used in previous studies that examined the structure of the CEBQ [14–16, 18]. Unlike those studies that were exploratory in nature, the current study aimed to investigate the feasibility of the existing structure in a new population, using a Chinese urban preschooler sample. Therefore, confirmatory factor analysis was adopted, which is commonly employed for cross-cultural test validation [12, 13, 17].

### Child factors related to eating behaviours

Child eating behaviours are associated with characteristics such as gender, age, and body mass index (BMI). Boys scored higher on fussy eating, emotional overeating, and food responsiveness than girls, but lower on enjoyment of food and food fussiness [14, 18]. Food responsiveness and enjoyment of food showed an increase with age, whereas satiety responsiveness, slowness in eating, emotional undereating, and desire to drink decreased with age in the UK [8]. On the contrary, in a Swedish sample, food responsiveness/emotional overeating and enjoyment of food have been found to significantly decrease with age, and food fussiness increased with age [15].

The associations between the CEBQ subscales and child BMI have shown relatively consistent patterns. Child BMI was negatively associated with the food avoidant subscales and positively associated with the food approach subscales [7, 16]. For instance, child BMI was found to be negatively correlated with satiety responsiveness and slowness in eating [11–14, 17], as well as with emotional undereating [14, 17] and food fussiness [7, 19]. Child BMI was positively correlated with enjoyment of food and food responsiveness [11, 14, 19], and emotional overeating [11, 13]. A few studies have found no significant correlations between child BMI and any CEBQ subscales [15, 18]. Unfortunately, the associations between child BMI and the CEBQ subscales of Chinese preschool children’s eating behaviours remain largely unknown.

### The present study

We aimed to (1) conduct confirmatory factor analysis on the CEBQ in a Chinese urban preschool children sample; (2) evaluate the measurement invariance and compare the latent means between child genders; (3) examine the associations between CEBQ factors and child age and BMI.

## Methods

### Participants and procedures

The study protocol was reviewed and approved by the Ethics Committee of College of Early Childhood Education, Capital Normal University, China. Participants were recruited from preschools in three urban districts in Beijing. Recruitment flyers and questionnaires were distributed by the preschool teachers to the families. Participants were asked to provide written informed consent and complete the hard-copy questionnaires, which were collected by the preschools.

The initial sample included 389 mothers, each with one preschool child (*Mage* = 4.49 years, *SD* = 0.58; 181 girls and 208 boys), residing in Beijing, China. Seventeen participants were removed due to careless or inconsistent response patterns. Specifically, 7 participants had 15 or more consecutively identical responses; 1 missed 17 items; and 9 endorsed both items in at least one of the contradictory item pairs (Pair 1: Q6 “*My child eats slowly*” and Q8 “*My child finishes his/her meal very quickly*”; Pair 2: Q29 “*My child eats less when s/he is angry*” and Q33 “*My child eats more when annoyed*”^1^) with 4-often or 5-always. The final sample consisted of 372 mothers with 197 boys and 175 girls (*M*age = 4.50 years, *SD* = 0.57; See Table 1).

**Table 1:** Demographic and BMI characteristics of the sample (*n* = 372)

	Mean (SD) / N (%)
Child gender	
Boys	197 (52.96%)
Child age	4.50 years (0.57)
Child BMI	15.76 (1.67)
Children’s weight categories	
Underweight (<5^th^)	11 (2.98%)
Normal weight (5^th^ ≤ BMI < 85^th^)	280 (75.88%)
Overweight (85^th^ ≤ BMI <95^th^)	41 (11.11%)
Obese (≥95 ^th^)	37 (10.03%)

### Measures

#### Children's Eating Behaviour Questionnaire

The CEBQ is a 35-item parent-report questionnaire rating on a five-point Likert-type scale (1 = never, 2 = seldom, 3 = sometimes, 4 = often, and 5 = always). Eight subscales (factors) of the CEBQ include Satiety Responsiveness (SR, 5 items, e.g., “*My child gets full up easily*”), Slowness in Eating (SE, 4 items; “*My child eats slowly*”), Food Fussiness (FF, 6 items; “*My child refuses new foods at first*”), Food Responsiveness (FR, 5 items; “*My child’s always asking for food*”), Enjoyment of Food (EF, 4 items; “*My child enjoys eating*”), Desire to Drink (DD, 3 items; “*If given the chance, my child would always be having a drink*”); Emotional Undereating (EU, 4 items; “*My child eats less when s/he is upset*”), and Emotional Overeating (EO, 4 items; “*My child eats more when worried*”). The original English version of the CEBQ was translated into Chinese, which was administered in the current study [20].

#### Child weight and height

Child weight and height were measured by preschool nurses at preschools (Table 1). BMI (kg/m^2^) was calculated based on child height and weight, resulting in a range (*M* = 15.76, *SD* = 1.67) from 11.89 to 26.50.

### Analyses

Confirmatory Factor Analysis (CFA) was conducted using the LAVAAN package in R [21]. We used the default ML (maximum likelihood) estimator, and adopted the full information maximum likelihood approach to the missing values. Model fit was evaluated based on goodness-of-fit indices of CFI and RMSEA primarily. As suggested by Hu and Bentler [22], the cut-off criteria for acceptable model fit are .90 for CFI and .06 for RMSEA. Factor scores were estimated using the method of regression. All factor loadings and covariances reported below are fully standardised.

Measurement invariance was investigated via multiple-group CFA. A series of models were specified and evaluated, starting with single-group analyses followed by multiple-group models. Specifically, the configural invariance model laid out the same structure for the two groups. The subsequent metric invariance model constrained corresponding factor loadings between the two groups. Additional constraints on the intercepts were posed in the scalar invariance model. If all models exhibited acceptable model fit and no significant difference in the chi-square statistics between consecutive models was observed, we could claim that the respective measurement invariance was established. Chen suggested that goodness-of-fit indices were sensitive to lack of measurement invariance [23]. She recommended the following cut-off points: When sample size is small (total *N* < 300) and the sample sizes are unequal, a change of 0.005 in CFI supplemented by a change of 0.010 in RMSEA would indicate noninvariance; when the sample size is adequate and sample sizes are equal across the groups, a change of 0.010 in CFI supplemented by a change of 0.015 in RMSEA would indicate noninvariance. With scalar invariance as prerequisite, we would be able to reliably compare the latent means between the two groups.

## Results

### Aim 1: CFA results

As shown in Table 2, the original model (Model 1) with 8 subscales as described in Wardle et al. revealed a poor model fit (CFI = .850 and RMSEA = .071) [8]. All items except item 15 (“*My child is difficult to please with meals*”; λ = − 0.094, *p* = .105) had significant factor loadings. Subsequently, item 15 was removed from the model. The goodness-of-fit indices of Model 2, although still not acceptable, showed slight improvement (CFI = .875 and RMSEA = .066). All factor loadings were significant, with two items below .40 (items 1 and 27 at .390 and .360, respectively).

**Table 2:** Model fit indices for the Chinese CEBQ

Model	Chi square	*df*	*p*	CFI	RMSEA
Model 1	1531.93	532	.000	.850	.071
Model 2	1311.35	499	.000	.875	.066
Model 3	1117.85	467	.000	.896	.061
Model 4	1086.99	466	.000	.900	.060
Model 5	1069.92	465	.000	.903	.059

*Note*. Reverse coded items: Q02, Q08, Q13, Q14, Q15

We continued to examine the modification indices and discovered that the top two suggestions for modification were both related to item 2 (“*My child has a big appetite*”). Improvement in model fit was expected if cross loadings were allowed for item 2 on factors food responsiveness and factor enjoyment of food. After interviewing several parents who took part in the survey, we concluded that the word “*appetite*” in Chinese was confusing. Therefore, item 2 was deleted from the model. The goodness of fit indices for Model 3 approached being acceptable, with CFI of .896 and RMSEA of .061.

The model fit was further improved by allowing within-factor error covariances, specifically, between item 17 (“*my child always has food in mouth if given the chance*” and item 18 (“*my child eats most of the time if given the choice*”) in Model 4 and a second error covariance between item 28 (“*my child eats less when upset*”) and item 29 (“*my child eats less when angry*”) in Model 5. Each pair of items was within the same factor describing similar behaviours. As they were presented in close proximity, it would be difficult for the participants to distinguish between these items, leading to resembling responses that could potentially account for the error covariances. The final model (Model 5) exhibited acceptable model fit with CFI above .90 and RMSEA below .06. Items 17 ad 18 were correlated at .549 and items 28 and 29 at .519. Table 3 shows the factor correlation matrix of Model 5. The factor loadings are presented in Table 4.

**Table 3:** Factor correlation matrix of Model 5^a^

	SR	SE	FF	FR	EF	DD	EU	EO
SR								
SE	.590***							
FF	-.092	-.126*						
FR	-.042	-.163*	.143*					
EF	-.392***	-.467***	.287***	.397***				
DD	.096	.039	.018	.522***	.112			
EU	.219**	.208**	-.029	.317***	-.039	.355***		
EO	.069	.072	.163**	.365***	-.004	.432***	.513***	

*Note*. *SR* Satiety responsiveness, *SE* Slowness in eating, *FF* Food fussiness, *FR* Food responsiveness, *EF* Enjoyment of food, *DD* Desire to drink, *EU* Emotional undereating, *EO* Emotional overeating

^a^Due to inverse factor loadings, FF should be conceptualised in the opposite direction

**Table 4:** Factor loadings of Model 5

Factor	Item	Loading
SR	Q1	.371
	Q2	–
	Q3	.709
	Q4	.821
	Q5	.500
SE	Q6	.882
	Q7	.805
	Q8	-.604
	Q9	.686
FF	Q10	.767
	Q11	.570
	Q12	.861
	Q13	-.495
	Q14	-.439
	Q15	–
FR	Q16	.709
	Q17	.820
	Q18	.796
	Q19	.689
	Q20	.648
EF	Q21	.817
	Q22	.903
	Q23	.877
	Q24	.735
DD	Q25	.669
	Q26	.790
	Q27	.367
EU	Q28	.761
	Q29	.778
	Q30	.807
	Q31	.483
EO	Q32	.811
	Q33	.912
	Q34	0.951
	Q35	.707

*Note*. *SR* Satiety responsiveness, *SE* Slowness in eating, *FF* Food fussiness, *FR* Food responsiveness, *EF* Enjoyment of food, *DD* Desire to drink, *EU* Emotional undereating, *EO* Emotional overeating

### Aim 2: measurement invariance between child genders

In order to investigate the measurement invariance between two genders, multiple-group CFA was carried out with gender as the grouping variable. The results are shown in Table 5. All single-group and multiple-group models showed marginally acceptable model fit. The differences in the chi-square statistics between the metric and configural invariance models and between the scalar and metric invariance models were not significant (*p*s = .11 and .41, respectively). The differences in the CFI and RMSEA were also not indicative of lack of measurement invariance. Therefore, it is concluded that the CEBQ achieved the scalar invariance, where the factor loadings, the intercepts, and the error covariances were constrained to be equal between the two groups, indicating no bias against either gender group.

**Table 5:** Model fit indices for the single- and multiple-group CFA models of the Chinese CEBQ between child genders

Model	Chi square	*df*	*p*	CFI	RMSEA
Male	823.16	465	.000	.891	.063
Female	768.25	465	.000	.900	.061
Configural	1591.41	930	.000	.896	.062
Metric^a^	1627.78	957	.000	.894	.061
Δ(Metric-Configural)	36.36	27	.11	.002	.001
Scalar^a^	1653.78	982	.000	.894	.061
Δ(Scalar-Metric)	26.00	25	.41	.000	.000

*Note*. ^a^error covariances were constrained between the two groups

When the two gender groups were compared on the latent means, the results in Table 6 showed that there was no significant difference in any factor. The largest difference was observed in the factor food fussiness, suggesting that girls were less fussy with food than boys, though the difference did not reach statistical significance (*p* = .085).

**Table 6:** Latent means of the girls in comparison to the boys

Factor	Latent mean	Std. error	*p*
SR	0.061	0.089	.495
SE	0.085	0.113	.450
FF^a^	0.178	0.103	.085
FR	-0.033	0.103	.749
EF	0.137	0.096	.152
DD	-0.120	0.092	.192
EU	0.089	0.079	.260
EO	-0.090	0.087	.297

*Note*. *SR* Satiety responsiveness, *SE* Slowness in eating, *FF* Food fussiness, *FR* Food responsiveness, *EF* Enjoyment of food, *DD* Desire to drink, *EU* Emotional undereating, *EO* Emotional overeating

^a^Due to inverse factor loadings, the factor FF should be conceptualised in the opposite direction. A high FF score indicates low food fussiness

### Aim 3: child age and BMI's associations with child eating behaviours

Eight factor scores based on Model 5 were derived from the CEBQ for each participant. All factor scores had a mean of 0. Their SDs ranged from 0.62 in emotional undereating to 0.94 in slowness in eating. We used factor scores instead of subscale total scores in the subsequent analyses. It is noted that the factor scores were highly correlated with the corresponding subscale total scores (i.e., all above .9). The Cronbach’s alphas are also presented in Table 7. Except for the subscales of satiety responsiveness and desire to drink, all subscales obtained higher than .7 internal reliability.

**Table 7:** Statistics of the subscale total scores and their reliabilities

Factor	*M*	*SD*	*r*^a^	Cronbach’s alpha
SR (*N* = 368)	11.40	2.71	.93	.67
SE (*N* = 372)	11.45	3.63	.98	.83
FF (*N* = 370)	14.92	3.76	.95	.76
FR (*N* = 369)	11.52	4.31	.99	.86
EF (*N* = 369)	13.23	3.53	.99	.90
DD (*N* = 369)	6.40	2.16	.93	.62
EU (*N* = 367)	10.39	2.89	.97	.82
EO (*N* = 370)	7.67	3.16	.98	.90

*Note*. *SR* Satiety responsiveness, *SE* Slowness in eating, *FF* Food fussiness, *FR* Food responsiveness, *EF* Enjoyment of food, *DD* Desire to drink, *EU* Emotional undereating, *EO* Emotional overeating

^a^correlation with corresponding factor scores

As shown in Table 8, age was a significant predictor for food responsiveness (*p* < .01), desire to drink (*p* < .05), emotional undereating (*p* = .05), and emotional overeating (*p* < .01). The results suggest that older children tended to have higher food responsiveness and less desire for drink, showing higher control over their eating behaviours. Also, older children experienced less emotional overeating or undereating. Child BMI was significantly correlated with satiety responsiveness (*r* = -.18, *p* < .001), slowness in eating (*r* = -.25, *p* < .001), and enjoyment of food (*r* = .11, *p* < .05). Child BMI was further converted to the BMI-for-age Z score for each participant, following the algorithm provided by the World Health Organization [24]. The BMI-for-age Z score showed similar correlations with satiety responsiveness (*r* = -.17, *p* < .01), slowness in eating (*r* = -.24, *p* < .001), and enjoyment of food (*r* = .10, *p* = .05). All the other correlations were not significant.

**Table 8:** Linear regression of factor scores on age

Factor	Beta	Std. error	*p*
SR	-0.014	0.062	.820
SE	0.036	0.085	.670
FF	-0.109	0.076	.151
FR	-0.202	0.076	.008^**^
EF	-0.071	0.077	.357
DD	-0.125	0.059	.036^*^
EU	-0.108	0.056	.054
EO	-0.226	0.071	.002^**^

*Note*. ^*^p < .05, ^**^p < .01

*SR* Satiety responsiveness, *SE* Slowness in eating, *FF* Food fussiness, *FR* Food responsiveness, *EF* Enjoyment of food, *DD* Desire to drink, *EU* Emotional undereating, *EO* Emotional overeating

To account for the inter-factor correlations, we entered the eight CEBQ factors into a multiple linear regression of the BMI-for-age Z score. Three factors (slowness in eating, food fussiness, and emotional overeating) revealed significant slopes (see Table 9). Subsequently, a stepwise model selection by the Akaike Information Criterion (AIC) was performed and resulted in a model with four factors (slowness in eating, food fussiness, emotional undereating, and emotional overeating). The adjusted R square of the final model was .068, suggesting that only a small percentage of the variance could be explained by the CEBQ factors.

**Table 9:** Stepwise regression of the BMI-for-age Z scores on the CEBQ factor scores

Factor	Full model	Final model		
SR	-0.07	–		
SE	-0.34***	-0.32***		
FF	0.16*	0.12		
FR	0.01	–		
EF	-0.14	–		
DD	0.14	–		
EU	0.23	0.23		
EO	-0.30**	-0.24*		
intercept	0.26***	0.26***		

*Note*. ^*^p < .05, ^**^p < .01, ^***^p < .001

*SR* Satiety responsiveness, *SE* Slowness in eating, *FF* Food fussiness, *FR* Food responsiveness, *EF* Enjoyment of food, *DD* Desire to drink, *EU* Emotional undereating, *EO* Emotional overeating

## Discussion

The present study assessed the hypothesised 8-factor structure of the CEBQ in a Chinese urban preschool children sample, and the results suggested a modified 8-factor structure of the CEBQ provided acceptable model fit in our sample [8]. Internal reliability of the subscales was good with Cronbach’s alpha greater than .70 in the majority of the subscales, except satiety responsiveness and desire to drink (Cronbach’s alpha was .67 and .62, respectively). Moreover, our study examined measurement invariance between child genders, showing no measurement bias against any gender in the CEBQ. Overall, our results supported the construct validity of the 8-factor CEBQ model in our sample.

The factor correlation pattern showed that satiety responsiveness was moderately correlated with slowness in eating (*r* = .59), which is consistent with factor correlations found in previous studies [8, 12]. Some research combined satiety responsiveness and slowness in eating into one subscale to measure satiety sensitivity [25], but the combined 7-factor structure failed to replicate in a low-income American sample [13]. We retained the two factors separately in our analysis and the final 8-factor model achieved acceptable model fit, which may represent these two factors obtain theoretical distinction in the Chinese sample.

We examined the associations between the CEBQ factors and child age and BMI. We found that, consistent with Wardle et al., food responsiveness showed significant increase with age, whereas emotional undereating and desire to drink decreased with age [8]. It is also discovered that emotional overeating decreased with age. The literature on child emotional eating has mixed findings [25]. It is possible that preschool children showed less emotional eating when they developed emotional regulation skills during this developmental stage [26].

The regression model of child BMI on the CEBQ factors suggested low convergent validity in the Chinese urban sample. Previous studies often found food responsiveness and satiety responsiveness associated with child BMI, but we failed to find any significant associations in our sample [27]. In the final stepwise regression model, only slowness in eating and emotional overeating predicted child BMI. Slowness in eating is sometimes attached to satiety responsiveness to jointly predict child BMI, because slowness in eating is considered to trigger internal satiety cues during food intake [25]. Emotional overeating has been found to be positively associated with child BMI and predict more weight gain over time [27, 28].

In our final model of the CEBQ, two problematic items were removed. Q15 (“*My child is difficult to please with meals*”) in food fussiness was removed due to the insignificant factor loading. Although this item had a reasonable factor loading of .64 in the original development of CEBQ, it showed very low factor loading in Chinese and Indian immigrant samples in Australia [12]. It was also removed in a Chinese infant sample and in a Singaporean sample [17, 18]. Q2 (“*My child has a big appetite*”) in satiety responsiveness was removed due to its cross-loadings on both food responsiveness and enjoyment of food. Our post-hoc interview with some of the parents suggested that the key word “*appetite*” was confusing, possibly due to inaccurate translation. Furthermore, this item seemed to consistently show a low factor loading in previous CFA models in the Chilean, Dutch, and Indian Australian samples [11, 12, 14].

When using the CEBQ in Chinese, extra caution should be paid to item translation. For example, item Q8 (“*My child finishes his/her meal very quickly*”) was translated to “My child eats very quickly”, which is not entirely consistent with the original item. In Q29 (“*My child eats less when s/he is angry*”) and Q33 (“*My child eats more when annoyed*”), the words “*angry*” and “*annoyed*” were translated into the same Chinese word “*sheng qi”*, which only captured the meaning of anger. Thus, professional translation alone is not sufficient when adapting the CEBQ measure in a new culture. It is critical to ensure that measures have equivalent meaning across cultures in order to make reliable inferences in the comparison of child eating behaviours [29]. Our study represented an initial step towards understanding child eating behaviours in various cultural contexts through validating the CEBQ.

### Limitations and future directions

Several limitations need to be noted. First, our sample was drawn from Beijing, the capital and one of the major metropolitan cities in China. Thus, the generalisability of the findings beyond the current sample is unknown. Future studies need to draw samples from other urban cities. Second, there were two error covariances in Models 4 and 5. Due to the fixed order of items in the questionnaire, it is possible that participants were easily confused by the items with similar meanings. Future efforts can be made to randomise the items within or across the factors when distributing the CEBQ. Third, a lack of cultural adaptation in the process of translating the CEBQ measure has been noted in the current study, which adopted an existing Chinese version of the CEBQ. Future studies should make necessary modifications to the CEBQ when studying the Chinese population, according to suggestions provided above. Finally, the present study used a cross-sectional design which precluded inferences about causation. Even though we tested the associations between child eating behaviours and the BMI scres, longitudinal studies are warranted to ascertain causal directions.

## Conclusion

The present study validated the CEBQ in a Chinese urban sample of preschoolers and explored the associations between Chinese young children’s eating behaviours and child age and BMI. Understanding the children's eating behaviours is an important step towards prevention and intervention of childhood obesity. It is pivotal that measurement tools such as the CEBQ are adapted accurately within different cultures. Our study contributed to this endeavour by validating the CEBQ measure as a tool for examining preschool children's eating behaviours in a Chinese urban sample. Future efforts to prevent or intervene Chinese urban children's weight problems might benefit from a focus on their eating behaviours.

## Data Availability

The datasets used and/or analysed during the current study are available from the corresponding author on reasonable request.

## Footnotes

1 In the Chinese version of the CEBQ that was translated by Tang and Zhou (2016), “*eats*” in Q6 and “*finishes his/her meal*” in Q8 were both phrased as “吃饭Chi fan”, rendering the two items exactly opposite in the meaning to each other. Similarly, Q29 and Q33 both used the word “生气 Sheng qi” to indicate “*angry*” and “*annoyed*”, resulting in another contradictory item pair.
